# The impact of ventilation rate on reducing the microorganisms load in the air and on surfaces in a room‐sized chamber

**DOI:** 10.1111/ina.13161

**Published:** 2022-11-18

**Authors:** Waseem Hiwar, Marco‐Felipe King, Harith Kharrufa, Emma Tidswell, Louise A. Fletcher, Catherine J. Noakes

**Affiliations:** ^1^ School of Civil Engineering University of Leeds Leeds UK; ^2^ Informatics and Telecommunications Public Company (ITPC) MOC Mosul Iraq

**Keywords:** aerosol transmission, AMPAS, bioaerosols deposition, infection mitigation, *Staphylococcus aureus*, surface decontamination, ventilation rate

## Abstract

Hospital‐acquired infections (HAIs) are a global challenge incurring mortalities and high treatment costs. The environment plays an important role in transmission due to contaminated air and surfaces. This includes microorganisms' deposition from the air onto surfaces. Quantifying the deposition rate of microorganisms enables understanding surface contamination and can inform strategies to mitigate the infection risk. We developed and validated a novel Automated Multiplate Passive Air Sampling (AMPAS) device. This enables sequences of passive deposition samples to be collected over a controlled time period without human intervention. AMPAS was used with air sampling to measure the effect of ventilation rate and spatial location on the deposition rate of aerosolized *Staphylococcus aureus* in a 32 m^3^ chamber. Increasing the ventilation rate from 3 to 6 ACH results in a reduction of microbial load in the air and on surfaces by 45% ± 10% and 44% ± 32%, respectively. The deposition rate onto internal surfaces λ_d_ was calculated as 1.38 ± 0.48 h^−1^. Samples of airborne and surface microorganisms taken closer to the ventilation supply showed a lower concentration than close to the extract. The findings support the importance of controlling the ventilation and the environmental parameters to mitigate both air and surface infection risks in the hospital environment.

**Practical Implications**

• Introducing a novel automated multiplate passive air sampler (AMPAS) to perform a pre‐programmed unattended sampling process enables the investigation of microorganism deposition rate over time in both a controlled environment and real‐world world environment that cannot otherwise be sampled due to the presence of vulnerable patients, contagious diseases, or other factors that prevent human intervention.

• Ventilation can mitigate the infection risk through both air and surface contamination in the indoor environment

• This study provides a realistic range of values for the loss rate of pathogens due to deposition onto surfaces for use in infection risk models.

## 1 INTRODUCTION

Hospital‐acquired infections (HAIs) remain a serious problem for healthcare providers worldwide and are further compounded by the ongoing growth of antibiotic resistance among microorganisms, which leads to rising death rates, durations of hospital stay, and treatment expenses. In 2017, HAIs were responsible for around 22 800 deaths, and the treatment of HAI patients in England alone cost £2.1 billion.^1^ Alongside long‐standing concerns over bacterial and fungal infections, the COVID‐19 pandemic has raised awareness of the significance of the built environment in the spread of communicable viral infections. Thus, controlling the environment, particularly ventilation, and maintaining hygienic surface conditions can have a significant impact on pathogen exposure and can lead to reducing infection risks.^2^

The importance of managing surface contamination for reducing transmission of infection in hospitals has been a topic of considerable interest in recent years. Studies show that when the surface bioburden increases, so does the risk of HAIs.^3^ Both hand contact and the deposition of microorganisms from the air can cause this surface contamination. The surface cleaning regime and hand hygiene compliance have been examined, and both were found to minimize surface contamination and consequently, infection risk.^4^, ^5^, ^6^ A review by Otter et al.^7^ indicated that there is growing evidence that contaminated surfaces are important for the transmission of several HAIs, including *C. difficile*, MRSA, *A. baumannii*, *P. aeruginosa*, and norovirus, and that outbreaks are better controlled when greater attention is paid to environmental decontamination. Dancer (2004)^8^ suggested that quantitative measurement of surface bioburden in a hospital could be used as a hygiene standard to manage infection risk, with a proposed aerobic colony count of <5 cfu.cm^−2^ suggested as indicative of an appropriate level of cleanliness.

Although microorganisms deposited on surfaces are recognized as a route to contamination, the factors which influence deposition have not been thoroughly addressed, and there is currently a lack of evidence about the contribution that deposited microorganisms make to infection risk. For many diseases, it is difficult to assess the relative importance of direct airborne (inhalation) exposure compared with indirect surface transmission, yet the two are related, as it is evident that airborne microorganisms deposit onto surfaces. Previous work shows that passive air sampling results can show the relation between air contamination and surface contamination and could thus possibly be used as a proxy for infection risk.^3^ Pasquarella et al. (2000)^9^ provide a detailed overview of active and passive air sampling and conclude that although passive samples cannot differentiate between different sizes of particles that deposit from the air, they can still provide a useful quantitative assessment of the microbial burden in the air as well as the contribution that microorganisms in air make to surface contamination. Although strategies such as improved ventilation and the addition of air cleaning devices have been found to affect the microbial bioburden in air^10^ and to reduce airborne transmission,^2^ there is limited evidence that these strategies can also decrease the hazard of transmission through surfaces.

To evaluate the relationships between the bioburden in air and deposition onto surfaces, it is necessary to undertake active and passive air sampling simultaneously, which can allow the calculation of deposition rates. Previous work has reported a 0.10–0.80 (ℎ^−1^) loss rate due to the deposition of dry (non‐biological) particles 0.55–1.91 μm in diameter onto surfaces at varying fan speeds (0, 5.4, 14.2, and 19.1 cm.s^−1^) in a laboratory room of 14.2 m^3^ volume.^11^ Another study that was conducted in a Class II biological safety cabinet of size 0.07 m^3^ and nebulized *Staphylococcus aureus* (*S. aureus*), has considered varying ventilation rates and sampling locations and found that the loss rate due to deposition of *S. aureus* onto surfaces is 0.14 h^−1^ at a ventilation rate of 1.7–18 ACH.^12^ Previous studies have also used passive sampling to measure the spatial variation in the deposition under a few different room geometries in comparison with a computational fluid dynamics (CFD) model using *S. aureus* in a biological chamber of size 32 m^3^.^13^ In the hospital, Wong et al.^14^ performed active and passive sampling at the same time and found that the loss rate due to deposition of the total aerobic count was 2.77 h^−1^ in the microbiological office and 5.5 h^−1^ in the intensive care unit.

The passive sampling technique is essential in this research; however, it provides an aggregate sample over a period of time. The variation of deposition rate with time in these previous chamber studies was not investigated due to the lack of a device to collect the microorganisms at intervals of time during the whole experiment. For this reason, the results could not show the full time‐cycle curve leading to a certain concentration or measure variability during steady‐state conditions. This is expected because it is difficult to capture the transient effects without employing an automated method. According to our knowledge, there is no commercial equipment that can expose the settle plates to the air for a defined period before covering them. In this study, we propose and test a new approach, the Automated Multiplate Passive Air Sampler (AMPAS), for remotely measuring microbial deposition over time. The contributions of this paper include (i) developing and testing a novel configurable device that can expose a settle plate to air for a pre‐determined interval, cover it, and autonomously expose a different one and (ii) investigating the effect of ventilation and spatial location on the deposition rate of microorganisms on surfaces in a controlled mechanically ventilated chamber setting.

## 2 METHODOLOGY

### 2.1 AMPAS device

#### 2.1.1 Concept, design, and components

**FIGURE 1:**
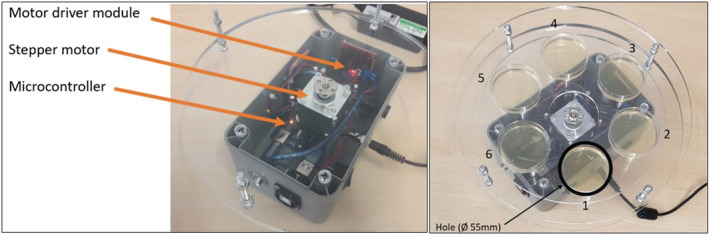
Automated multiplate passive air sampling device and components.

The primary goal of the AMPAS device is to enable sampling for several discrete time intervals over a defined period. The device comprises a series of six Petri dishes arranged in a circle, covered by a rotating tray controlled by a stepper motor (Figure 1). The device is programmed to expose each agar plate to the microorganisms in the air at pre‐determined times and for pre‐programmed periods before covering them, without human intervention, to ensure they are no longer exposed to air.

The main electronic and mechanical components of the device are the microcontroller, stepper motor, motor driver module, power source, box container, and circular trays. The microcontroller (Elegoo mega 2560) was controlled by a C program to manage the operation of the device and set the timers and actuators. The NEMA 17 bipolar stepper motor was used to rotate the middle trays at steps of 0.9°. The motor driver module (Neuftech L298N Dual Channel H‐Bridge) was used to convert the received signal by alternating the polarity according to the instructions of the microcontroller to decide the speed and number of steps, and the direction of each step. The Perspex trays include a base tray of Ø 260 mm circular sheet with a hole in the center to allow the motor shaft through. This tray protects the other elements from contamination, and the motor is fixed to it. The middle two trays of Ø 210 mm circular sheets are connected to each other to construct six compartments to hold the Ø 55 mm agar plates intended for exposure/protection, forming the rotating part of the device. Finally, the upper tray of the Ø 260 mm circular sheet is connected to the base tray, and it has one Ø 55 mm hole to expose one plate at a time while protecting the others from exposure.

#### 2.1.2 Initial testing and validation

Initial safety testing was performed by the electronic services workshop (Faculty of Engineering, University of Leeds) to ensure the safety and robustness of the device. The alignment of the plates and the hole above them was tested in the laboratory and found to be accurate with less than 0.37 mm bias. The stepper motor moves in steps of 0.9° (400 steps/360°), so the complete cycle of the AMPAS rotation (six plates) consists of 67, 66, 67, 67, 66, and 67 steps.

The timing of AMPAS air exposure was tested using a stopwatch for periods of 10, 60, 600, and 3600 s. The timing was accurate in all cases since it was controlled by a C‐function that provides timing accuracy to the millisecond.

### 2.2 Microbial experiments

**FIGURE 2:**
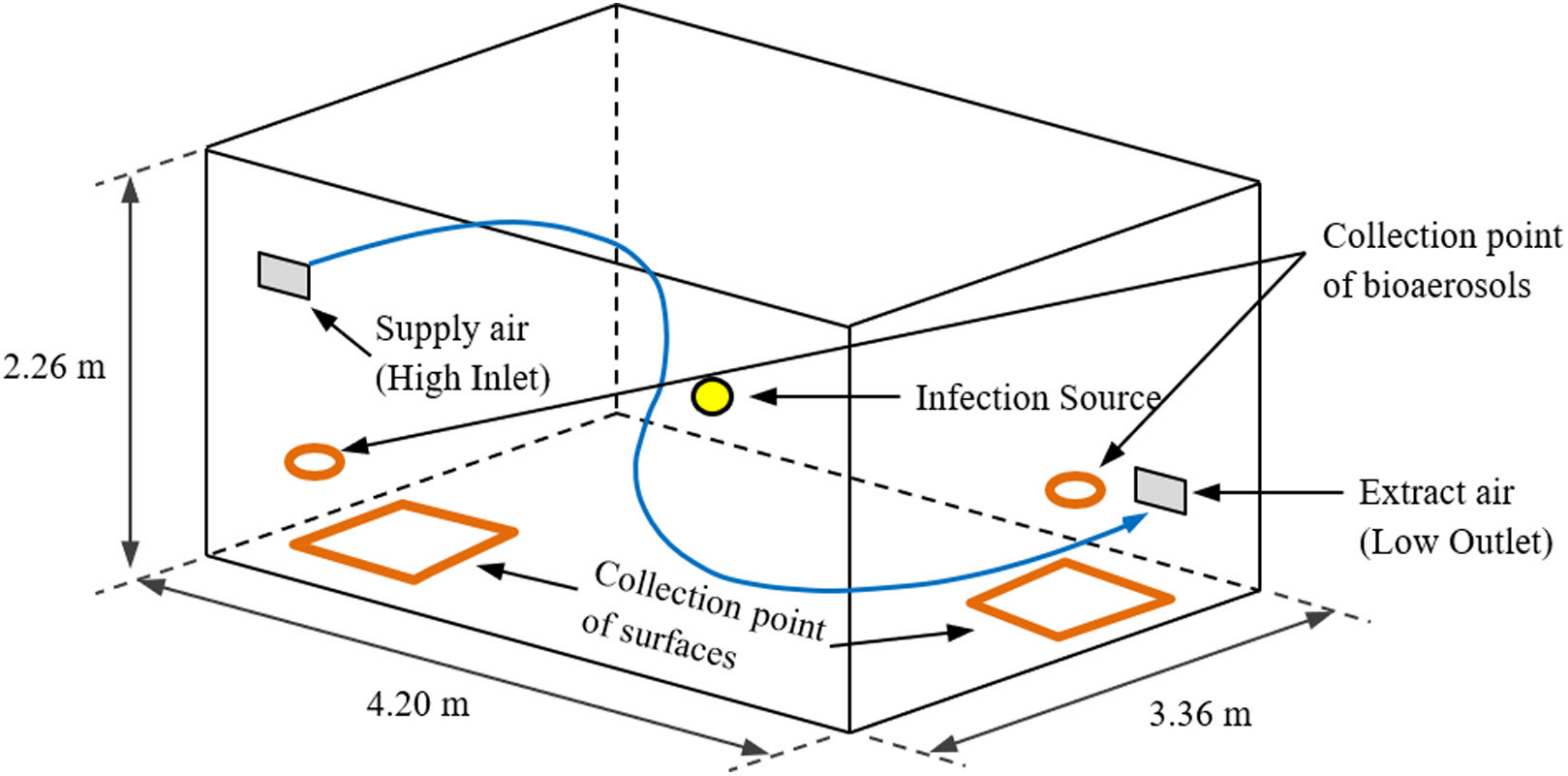
The aerobiological chamber dimensions and the collection points of air and surfaces.

The microbial experiments were all conducted in the controlled aerobiological chamber ~32 m^3^ (Figure 2) with the same ventilation regime (high grid inlet‐ low grid outlet) as in Eadie et al.^15^ Experiments were carried out at an airflow rate of 0.027 and 0.054 m^3^s^−1^, equivalent to three and six air‐changes‐per‐hour (ACH), respectively, and under a slight negative pressure (0.5 bar). The ventilation is HEPA filtered at the supply and the extract to provide contaminant‐free inlet air and ensure safe discharge. Previous research has demonstrated that the chamber ventilation facilitates good air mixing, so no mixing fans were used. Aerosolized *S. aureus* (ATCC 6538) suspended in distilled water was generated using a 6‐jet Collison nebulizer (BGI, USA) operating at 12 L.min^−1^, and released through a tube and into the center of the chamber to produce aerosols in the range of 0.3–5 μm diameter.^13^ Throughout all experiments, approximately 90% of *S. aureus* aerosols were found to be 0.65–1.1 μm in diameter (collected at stage 6 of the Andersen sampler) while around 10% were found to be 1.1–2.1 μm in diameter (collected at stage 5 of the Andersen sampler); the combined concentration of *S. aureus* aerosols that are 2.1–7 μm in diameter was less than 1% (collected at stages 1–4 of the Andersen sampler).^15^

The culture broth, suspension for nebulizing (~1 × 10^6^ cfu.ml^−1^), and the tryptone soy agar (TSA) media used in both air (Ø 90 mm plates) and surface sampling (Ø 55 mm plates) were all prepared following methods in King et al., 2013, and Eadie et al., 2022.^13^, ^15^ An initial pilot study found that there is 7% ± 2% of natural decay of *S. aureus* in the same environment and experiment time. The chamber was set at an ambient air temperature of 24 ± 1 °C, with a relative humidity of 50% ± 2%.

#### 2.2.1 Checking the negative control of AMPAS

**FIGURE 3:**
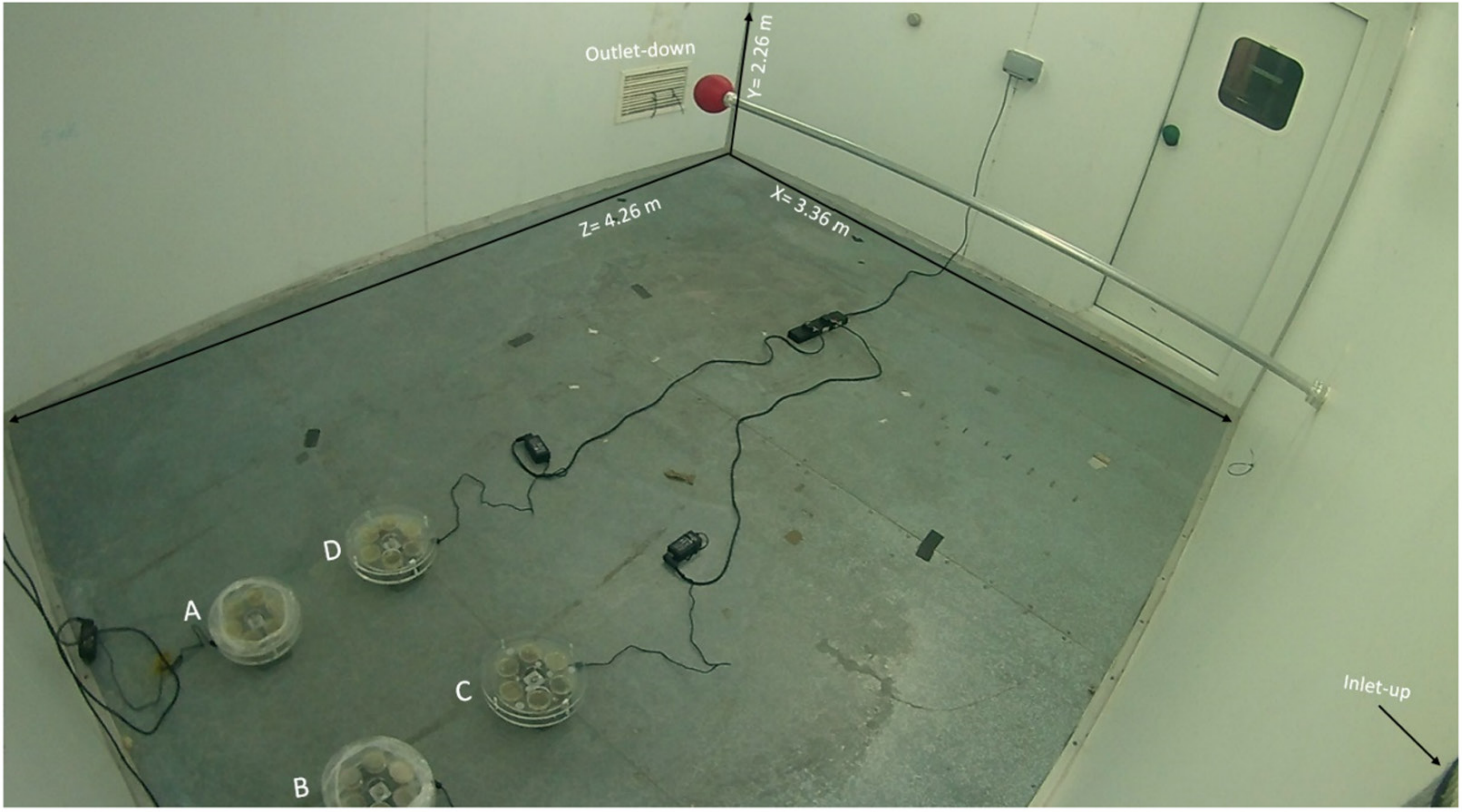
Automated multiplate passive air sampling placement in the controlled environment chamber.

In the design of AMPAS, it is assumed that when the test plate is exposed to air through the hole (positive control), the other five plates should be completely protected, and no microbial deposition should be detected on them, provided that the device does not rotate (negative control). Four AMPAS devices were put close together in front of the ventilation inlet and to the left of the nebulizer inlet, where high concentration and well‐mixed air are expected (Figure 3). The AMPAS devices were not operated in this experiment; plate 1 was exposed to air, and plates 2–6 were covered throughout the experiment. A continuous release of aerosolized *S. aureus* was introduced to the chamber for 165 min at 6 ACH. The experiment was replicated three times.

**FIGURE 4:**
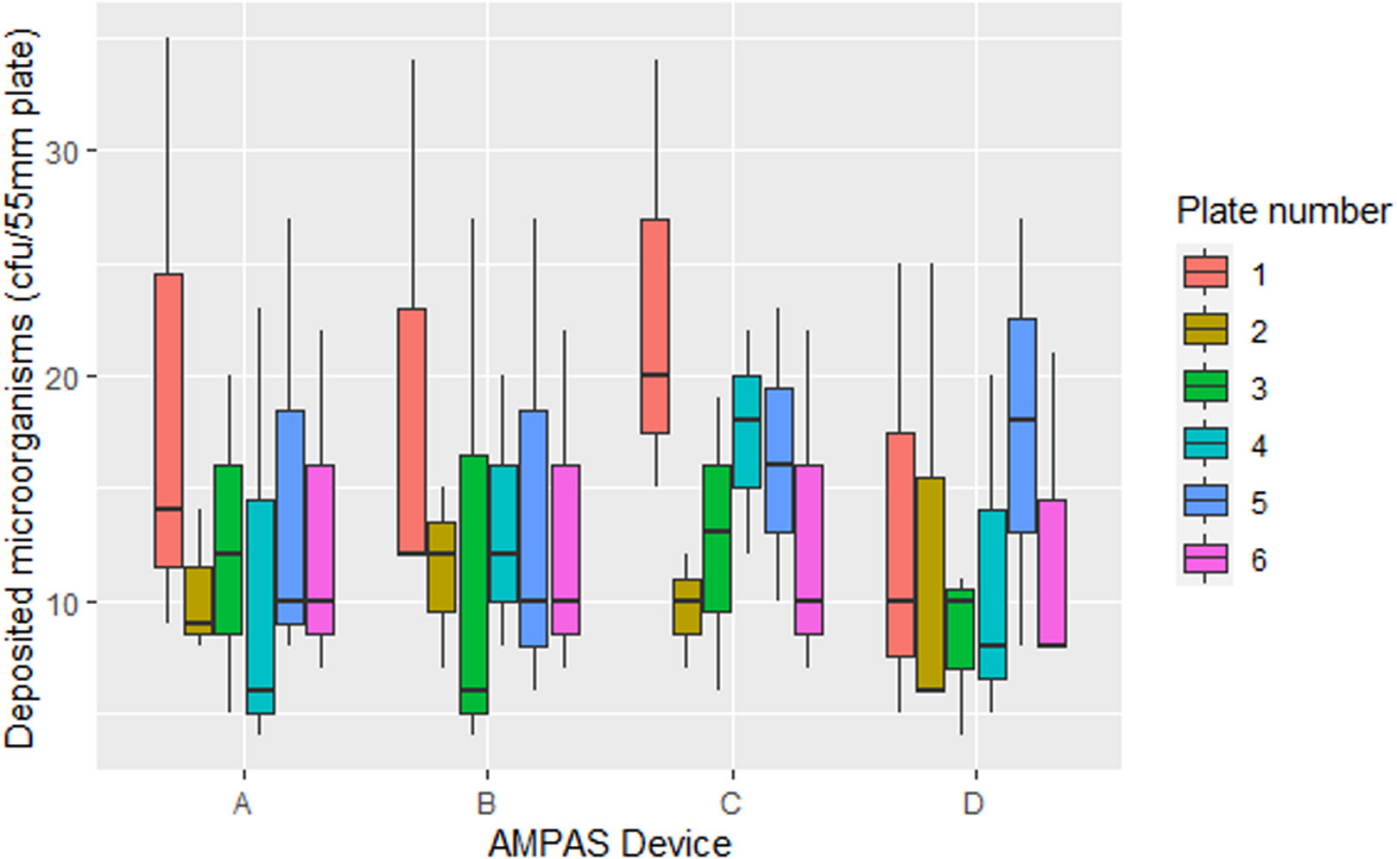
Sampling results from the negative control experiment before making improvements to the device.

The initial experiment (see results Figure 4) showed that during this controlled study, there was contamination on plates 2–6. To overcome the problem of undesirable contamination of protected plates, a number of alterations to the initial design were tested. A plastic wrap (Food cling film) was wrapped around the periphery of two of the AMPAS devices (A) and (B). The gap between the edge of the plate and the top tray was reduced from 5 to 1 mm in devices (A) and (C), while device (D) was used as the original design, without adjustments (no plastic wrap and 5 mm gap). The second set of tests was carried out to identify which design would provide higher protection to the covered plates; the experiment was replicated three times for validation.

#### 2.2.2 Checking the consistency of deposition rate onto the plates of AMPAS

Experiments were carried out to determine whether the AMPAS device measured consistently on all of the sample plates and all devices. The four AMPAS were placed inside the chamber in a similar placement to that of the previous experiment. The plates were numbered from 1 to 6, where plate number 1 is under the hole at the beginning, and plate number 2 is next to it. The rotation was clockwise from plate number 1 to plate number 6. The device was programmed to wait for 60 min (exposing plate number 1 for 60 min to air), rotate every 15 min (exposing plate number 2–6), then return to position number 1 and stop rotating.

#### 2.2.3 Measuring the impact of ventilation rate and spatial location on surfaces deposition

Experiments to measure deposition rates used both passive (AMPAS) and active air sampling. Two bioaerosol air sample collection points (Figure 2) were located near the air inlet and the air outlet at the same dimensions indicated by Eadie et al. (2022).^15^ In each experiment, the air was sampled five times for 4 min at an interval of 15 min using a six‐stage Anderson air sampler that was operated at a flow rate of 28 L.min^−1^. Surface samples were performed using four AMPAS devices placed together 50 cm from the air outlet and 60 cm from the adjacent wall, or 50 cm from the air intake and 60 cm from the adjacent wall. Sampling was carried out for 15 min, in 15‐min cycles and was repeated five times, recording the average value at each interval.

At each ventilation rate (3 & 6 ACH) and at each location (near the ventilation inlet and near the outlet), six experiments were conducted with five repetitions with varied time intervals under steady‐state conditions for each experiment, producing a total sample size of 120 values for air sampling and 120 values for surface sampling. For AMPAS, plate number 1 was excluded because it included the build‐up and decay states rather than the steady state alone, and in each experiment, the results from all four AMPAS devices exposed in the same time interval were collated and presented as a single value to increase the surface area of sampling.

Ventilation and nebulization commenced at the start of each experiment, and the first 60 min were employed to let the room achieve steady‐state conditions. Air and surface sampling then lasted 75 min with the nebulizer and ventilation operating continuously. Following sampling, the nebulizer was switched off, and the room ventilation rate was increased to 12 ACH for 30 min to flush any remaining airborne microorganisms from the room. Following the experiment, the plates were incubated at 37°C for 24 h. A correction table (appendix B—400 Hole Count) was used to apply positive hole correction for the air samples to correct for potential over‐counting under higher bioaerosol concentrations.^16^

The percentage of bioaerosols load reduction and deposition microorganism rate through the effect of changing ventilation from 3 to 6 ACH was calculated using Equation 1

(1) $$R_{l}=\frac{\sum_{1}^{i}\left(\frac{m_{3,l}-n_{i,6,l}}{m_{3,l}}\right)}{i}\times 100\%$$

Where, $R_{l}$ is the percentage of reduction in a specific location (inlet or outlet), i is the number of samples of data, $m_{3,l}$ is the mean of bioaerosols load or deposited microorganism load in a specific location (inlet or outlet) at 3ACH, $n_{i,6,l}$ is the single data value at the same location *l* for sample i at 6ACH. For example, at the inlet location (L) for bioaerosols load, i equals to 30, m_3,l_ equals to 3797 cfu.m^−3^, $n_{i,6,l}$ equals to is an array of 30 values ($n_{1,6,l},n_{2,6,l}\ldots .n_{i,6,l}$).

### 2.3 Statistical analysis

R version 4.2.0 was used to process data and plot the graphs.^17^ A one‐way analysis of variance test was conducted to evaluate the separate hypotheses that no difference existed between sampling location or device design. A significance level of 0.05 was used throughout.

## 3 RESULTS

### 3.1 The negative control of AMPAS

The initial experiment showed that although plate 1 (the test plate) generally had higher deposition, all plates had similar concentrations of *S. aureus*, indicating that the microorganisms from the air are depositing on the plates even when they are assumed to be protected (Figure 4). This shows that deposition is complicated, with air movement through the sampler device enabling deposition onto plates that were not open vertically to the air. As well as demonstrating that the device required modification it also illustrates that microbial contamination of surfaces that do not appear to be directly exposed can happen with low velocity airflows created only by a ventilation system; this may have implications for contamination of other complex devices in clinical settings.

### 3.2 The final AMPAS design

**FIGURE 5:**
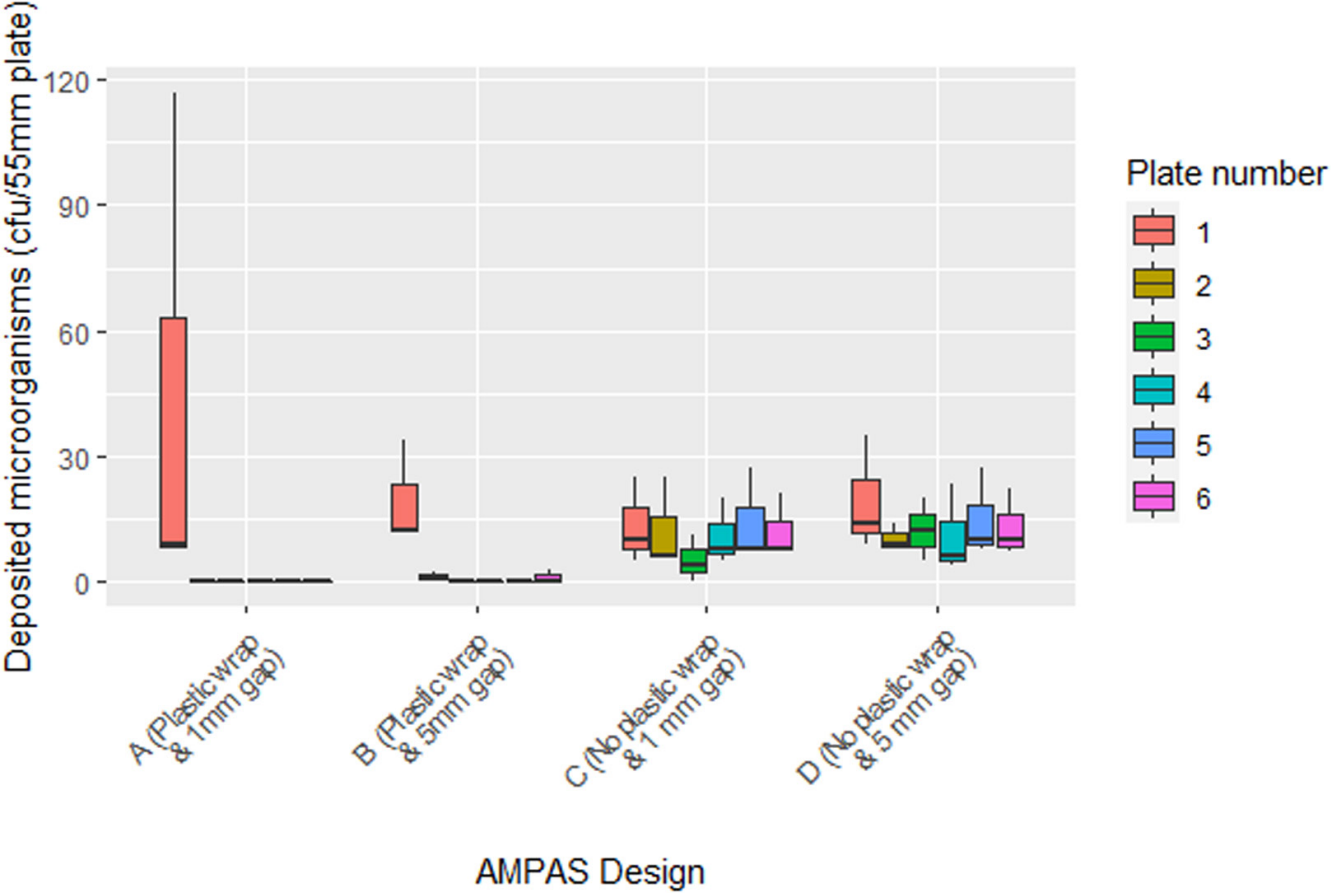
Automated multiplate passive air sampling negative control experiment in the chamber with four different designs A–D.

The use of plastic wrap around the perimeter of the AMPAS device (A and B) had the most significant effect and, together with reducing the gap to 1 mm (A) significantly (*p* < 0.0001), improved the reliability of the collected data and eliminated the problem of undesired contamination (Figure 5).

### 3.3 Checking the consistency of deposition rate onto the plates of AMPAS

**FIGURE 6:**
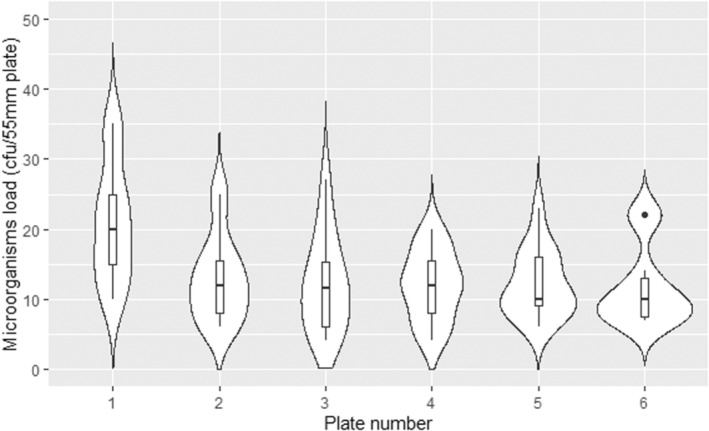
Comparison of the deposited microorganisms on six plates.

The average deposited microorganisms on plates 2–6 showed no significant difference while plate number (1) had a higher deposition, due to being exposed to air for a more extended period at the beginning and the end of the experiment Figure 6.

**FIGURE 7:**
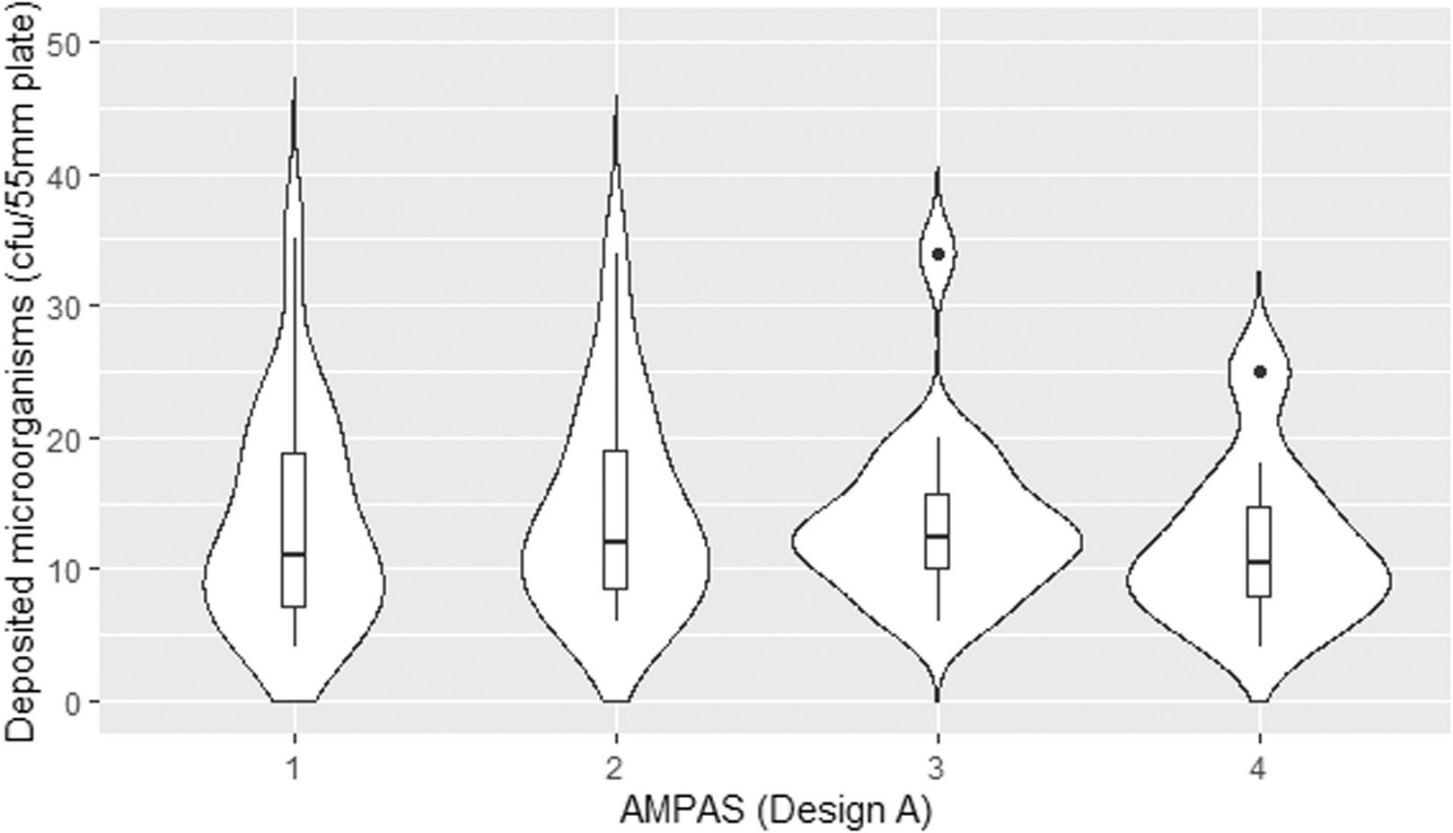
Comparison of the deposited microorganisms on four AMPAS devices (1 mm gap with plastic wrap) under steady state conditions.

As shown in Figure 7, different devices with the same design and settings had no significant difference in the deposition of microorganisms. This confirms that using the AMPAS device provides consistent results over time under steady‐state conditions, and that there is no significant difference (*p* > 0.5) between the four devices used in the study.

### 3.4 The impact of ventilation rate and location on surfaces bioburden

Table 1 shows the descriptive statistics of the bioaerosols load in air and deposition rate of microorganisms on surfaces, under steady‐state conditions across all of the experiments. The comparison between the values measured near the ventilation inlet and the values measured near the outlet was based on different experiments as it was not possible to measure in both locations simultaneously. As predicted, the experiments carried out at a ventilation rate of 6 ACH resulted in a reduction of the airborne microorganism concentration compared with 3 ACH. The reduction was calculated as a percentage for all experiments to show the difference in concentration from experiments at a ventilation rate of 3 ACH. At a ventilation rate of 3 ACH, the measured bioaerosols load near the inlet and the outlet were 3797 ± 426 cfu.m^−3^ and 5599 ± 565 cfu.m^−3^, respectively. At 6 ACH under the same experimental conditions, the bioaerosols load near the inlet and the outlet were lower at 2218 ± 350 cfu.m^−3^ and 3167 ± 580 cfu.m^−3^, respectively. Using Equation 1, the percentage of reduction in bioaerosols load when operating ventilation at 6 ACH compared with 3 ACH was found to be 43% ± 8% near the inlet and 45% ± 10% near the outlet.

**TABLE 1:** Descriptive statistics of bioaerosol concentration and deposition rate of microorganisms on surfaces.

ACH	Air sampling collection point	Bioaerosols load (cfu.m−3)		Deposited microorganism load (cfu.m^−2^.h^−1^)	
		Mean ± SD (Min‐Max), *n* = sample size	Reduction percentage	Mean ± SD (Min‐Max)	Reduction percentage
3	Near supply air (Inlet)	3797 ± 426 (2878–4437), *n* = 30		3696 ± 1885 (758–7036), *n* = 30	
	Near extract air (Outlet)	5599 ± 565 (4376–6767), *n* = 30		9450 ± 4469 (4363–22 171), *n* = 30	
	Mean across both locations	4698 ± 1035 (2878–6767), *n* = 60		6573 ± 4470 (758–22 171), *n* = 60	
6	Near supply air (Inlet)	2218 ± 350 (1669–2861), *n* = 30	43% ± 8%	2442 ± 910 (505–4042), *n* = 30	33% ± 25%
	Near extract air (Outlet)	3167 ± 580 (1933–4571), *n* = 30	45% ± 10%	5086 ± 2961 (1011–10 611), *n* = 30	44% ± 32%
	Mean across both locations	2693 ± 674 (1669–4571), *n* = 60		3764 ± 2548 (505–10 611), *n* = 60	

The rate of microbial deposition on surfaces near extract air vent/grille was 9450 ± 4469 cfu.m^−2^.h^−1^ and near the inlet was 3696 ± 1885 cfu.m^−2^.h^−1^ at 3 ACH ventilation rate. For 6 ACH, the deposition rates near the outlet and the inlet were 5086 ± 2961 cfu.m^−2^.h^−1^ and 2442 ± 910 cfu.m^−2^.h^−1^, respectively. The percentage of the reduction in deposited microorganism load at 6 ACH compared with 3 ACH ventilation rate was found to be 33% ± 25% near the inlet and 44% ± 32% near the outlet.

**FIGURE 8:**
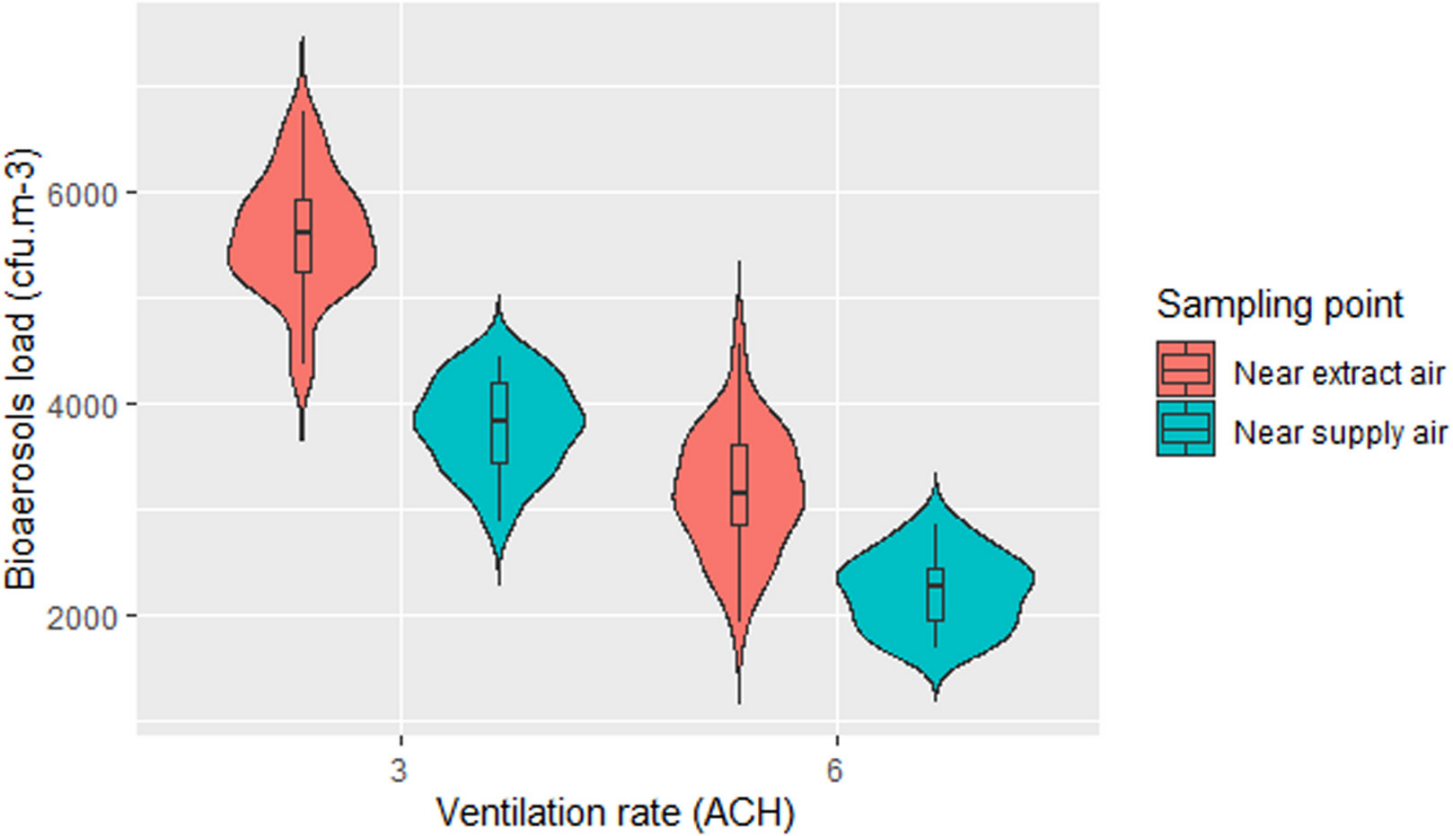
Airborne bioaerosol load under steady‐state conditions at 3 and 6 ACH ventilation rates and at two locations (ventilation supply and extract) in the chamber.

The bioaerosol concentration under the steady‐state conditions at 3 and 6 ACH ventilation rate pools the data from all five air samples in each of the six experiments at each condition. Figure 8 shows that the bioaerosol load near the ventilation extract (Outlet) was significantly higher (*p* ˂ 0.001) than the bioaerosol load near the supply (Inlet) at both 3 and 6 ACH ventilation rates. The results also shows that although there is variability in aerosol concentration at the same position, the differences are not significant. This confirms that the chamber has reached steady‐state conditions after the initial 60 min and remains relatively stable throughout all five samples.

**FIGURE 9:**
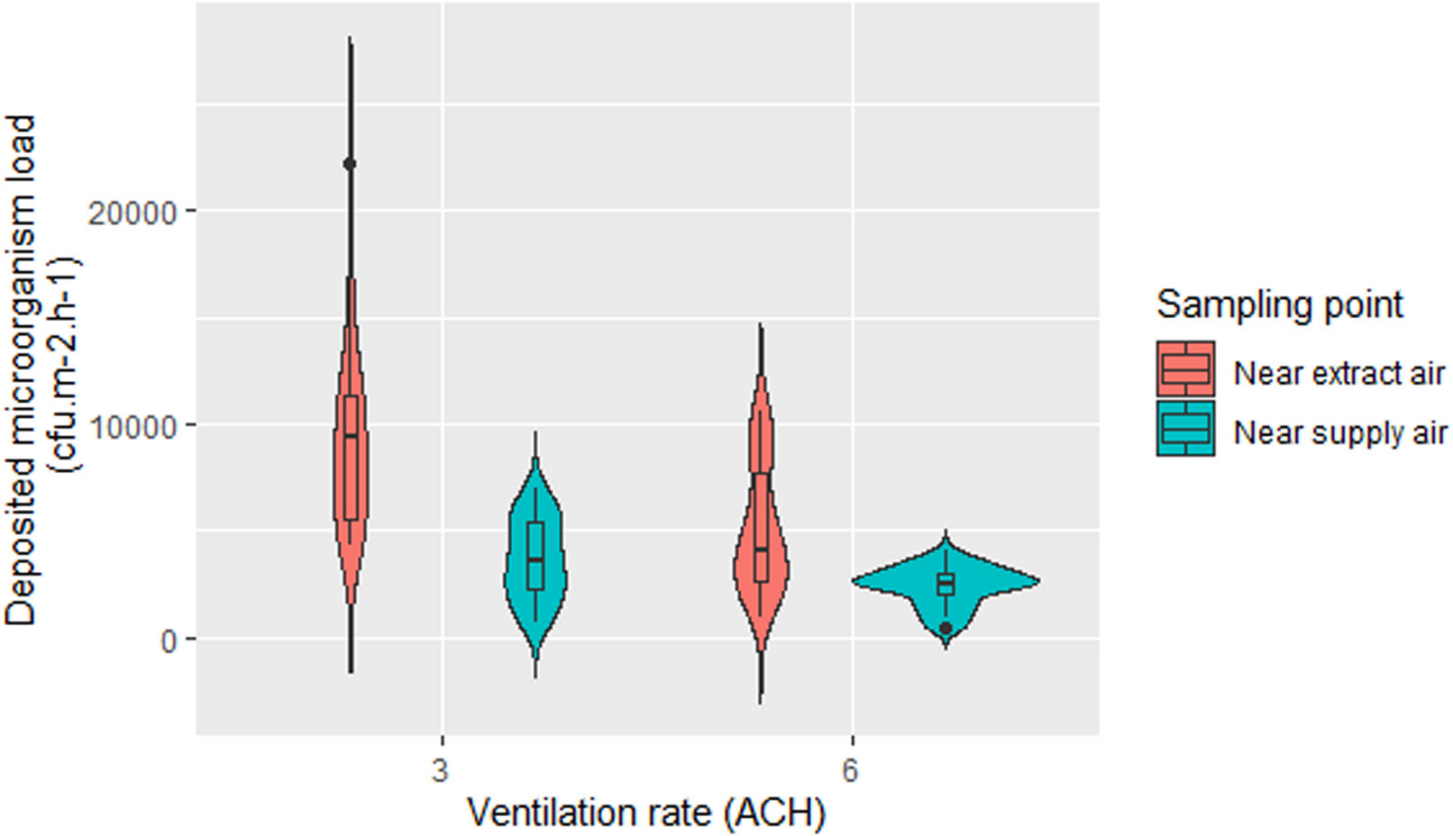
The mean deposited microorganisms load under the steady‐state conditions at 3 and 6 ACH ventilation rates sampled near the ventilation inlet and the outlet.

As shown in Figure 9, this same trend is also seen with the AMPAS surface samples. The deposited microorganisms load near extract air was significantly higher (*p* < 0.001) than the deposited load near the inlet at both 3 and 6 ACH ventilation rates.

**FIGURE 10:**
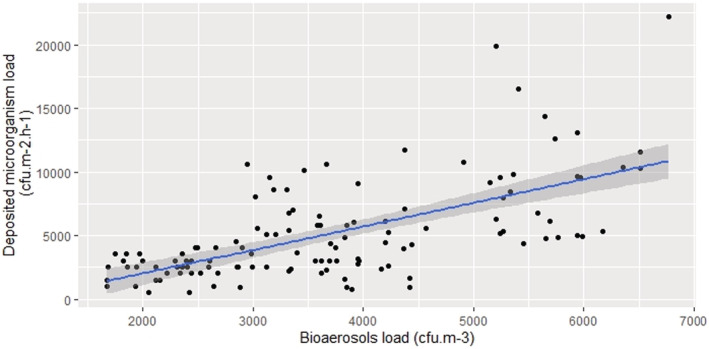
The relationship between deposited microorganism load (cfu.m^−2^.h^−1^) and bioaerosols load (cfu.m^−3^).

To further explore the relationship between microorganisms in air and on surfaces, data from samples at the same time point across all of the experiments was analyzed. The relationship between deposited microorganism load (cfu.m^−2^.h^−1^) and bioaerosols load (cfu.m^−3^) was a moderately significant positive correlation (*r* = 0.63 [95% CI = 0.51–0.72], *p* ˂ 0.01, 120) as shown in Figure 10. The concentration of microorganisms on surfaces inside the chamber can be calculated using a simple linear regression model (Equation 2).

(2) y=0.22x+2575

Where, *y* is the concentration of microorganisms on surface (cfu.m^−2^.h^−1^) and *x* is bioaerosols load (cfu.m^−3^). This equation (2) is only accurate when there is a high concentration of bioaerosols and in a controlled environment.

### 3.5 The loss rate due to deposition onto surfaces

Under the steady‐state conditions, the total loss rate due to deposition onto surfaces can be calculated as in Equation 3

(3) $$\lambda_{d}=\lambda_{d.f}+\lambda_{d.w}+\lambda_{d.c}$$

where, $\lambda_{d}$ is the loss rate due to deposition onto total inner room surfaces per hour. $\lambda_{d.f},\lambda_{d.w}\lambda_{d.c}$ are the loss rate due to deposition onto floor, wall, and ceiling surfaces per hour, respectively.

$\lambda_{d.f}$ can be calculated according to Equation 4

(4) $$\lambda_{d.f}=\frac{C_{\mathrm{sf}}A_{f}}{CV}$$

Where $C_{\mathrm{sf}}$ is the indoor deposited microorganisms' concentration on the floor ($\mathrm{cfu}.m^{-2}.h^{-1}$), as found using AMPAS. $A_{f}$ is the surface area of the floor ($m^{2}$), C is bioaerosols concentration (cfu.m^−3^) and V is the volume of the room ($m^{3}$).

The deposition rate of bioaerosols on the ceiling and walls was substituted by a percentage equal to 23% and 44% of the deposited microorganisms' concentration on the floor, respectively, based on Liu et al.^18^ Although this is an estimation and it allows for biased results because the inlet and outlet for ventilation for Liu et al.^18^ were located in the ceiling, which may change the surface deposition pattern. Assuming that the floor and the ceiling have the same surface area, Equation 5 uses the same principle as Equation 3 and calculates the loss rate due to deposition, taking into account the deposition onto the walls and ceiling as a percentage of the deposition on the floor (based on real data from the experiment).

(5) $$\lambda_{d}=\lambda_{d,f}+\beta_{w}\lambda_{d,f}\frac{2H\left(L+W\right)}{\mathrm{LW}}+\beta_{c}\lambda_{d,f},$$

**FIGURE 11:**
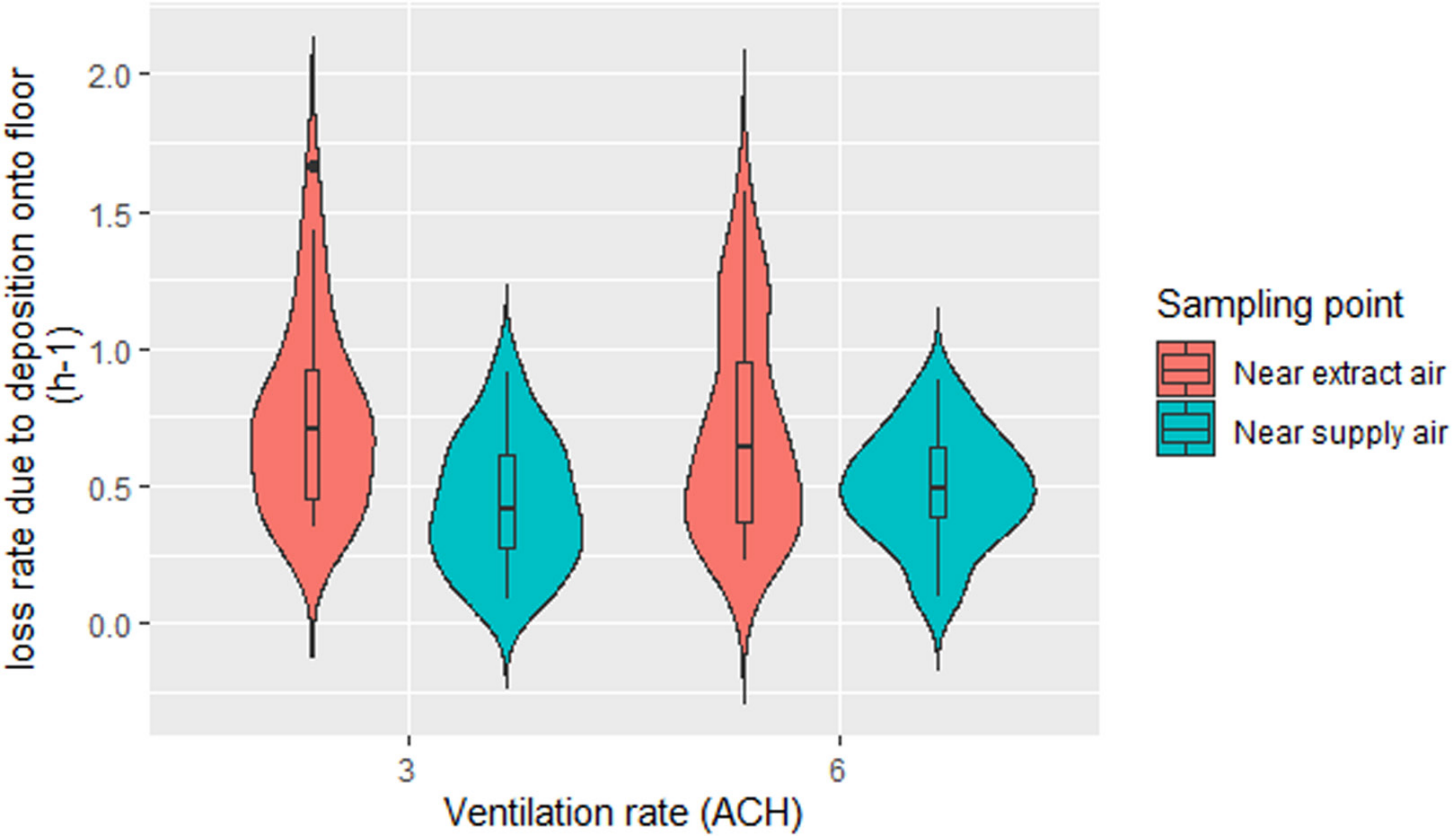
The loss rate due to deposition on the floor at different ventilation rates and locations.

Where, $\beta_{w}$ and $\beta_{c}$ are the percentages of $C_{\mathrm{sf}}$, which is the indoor deposited microorganisms' concentration on the floor ($\mathrm{cfu}.m^{-2}.h^{-1}$) for deposition on the walls and ceiling, respectively. The total loss rate due to deposition on surfaces ($\lambda_{d,f}$) was 0.6 ± 0.33 (h^−1^) (Figure 11) and all surfaces $\left(\lambda_{d}\right)$ was 1.38 ± 0.48 (h^−1^).

## 4 DISCUSSION

To the best of our knowledge, this is the first study to investigate the impact of ventilation rate on the deposition rate of microorganisms onto surfaces over time, using a novel passive sampling device in a room‐scale controlled environment under steady‐state conditions. The validation of AMPAS showed that bacteria were able to enter through the 5 mm gap in the initial design and contaminate agar plates that were protected by the upper tray. Reducing the gap to 1 mm did not significantly improve the protection because bacteria were still able to enter through the perimeter. Adding plastic wrap to protect the latter source of contamination significantly improved the protection and ensured no contamination in case of the 1 mm gap, and only slight contamination occurred on the plates adjacent to the uncovered hole in case of the 5 mm gap. Thus, the device was finalized with a 1 mm gap and plastic wrap around the perimeter (Figure 5). Furthermore, the deposition onto different plates of the same device and the average deposition of plates in different devices were shown to be consistent, as shown in Figures 6 and 7.

The results show that increasing the ventilation rate from 3 ACH to 6 ACH results in a reduction of bioaerosols load in the air by 43% ± 8% and 45% ± 10% when sampling near the inlet and the outlet, respectively. This result is close to the 50% reduction expected through the well‐mixed assumption that is typically used to estimate the impact of ventilation rate on contaminants in air. The mean reduction in our experiments is consistently slightly lower than 50% which may be due to the effects of air mixing patterns, sampling effects or that in the real‐world setting it is not possible to measure the ventilation rate to the same accuracy as a theoretical model.

The same trend was seen in the deposited data, with an increase in the ventilation rate reducing the deposited microorganism load by 33% ± 25% and 44% ± 32% when sampled near the inlet and the outlet, respectively. These values are a similar order of magnitude reduction with the increase in ventilation rate as seen in the air samples suggesting that the relative deposition rate remains fairly consistent with a change in ventilation rate under the conditions studied. The results show that there does appear to be more variability in the surface sample data than the air sample data.

Across all experiments, the concentration of microorganisms in the air and on surfaces near the extract air (Outlet) was significantly higher (*p* ˂ 0.001) than near the supply air (Inlet) at both 3 and 6 ACH ventilation rates. The loss rate due to deposition on the floor ($\lambda_{d,f}$) for both locations near the inlet and near the outlet was also investigated, which was found to be 0.60 ± 0.33 (0.09–1.69) h^−1^. This result agrees with the literature and confirms the influence of deposition loss rate on the bioaerosols load. However, to obtain more realistic data, the deposition on the walls and ceiling was also considered as a percentage of floor deposition to find the total deposition on all surfaces ($\lambda_{d}$) that is more realistic but slightly higher (1.38 h^−1^) than what the literature suggests. A previous work (Lai et al., 2012)^12^ has found that the deposition rate near the outlet was 1.53 and 1.79 times higher than near the inlet at a ventilation rate of 1.7 ACH and 10.3 ACH, respectively. It also confirms our results that the variability in aerosol concentration at different positions, even in a reasonably well‐mixed room, could be comparable to the difference that results from doubling the ventilation rate. This makes sense as fresh air supply affects the concentration of airborne microorganisms. Near to the ventilation supply grille, there is a greater influence from the clean air supplied to the room enabling a higher level of dilution. This observation highlights the need to consider the efficiency of ventilation techniques and regimes. The higher variation in the deposited microorganisms near the outlet is likely to be due to the positioning of the collection points. It can be clearly seen in Figures 8 and 9 that the extract air outlet is positioned lower than the fresh air inlet and closer to the collection point. This means that the airflow near the outlet is more disturbed than near the inlet, which may cause a higher variation in the results. Our experiments were all carried out with air supplied through a high‐level inlet and extracted through the low‐level outlet and we have not looked at the effect of other airflow patterns in this study. However, King et al., 2013, shows both through computational fluid dynamics and steady‐state deposition experiments that the flow patterns are significant promotors or mitigators for deposition. The relationships between ventilation flow pattern and deposition are complex and require further investigation.

In the real world, the loss rate due to deposition onto the floor surface has been found to be 5–10 times higher than in our experiments; it was 2.77 h^−1^ in the microbiological office and 5.5 h^−1^ in the ICU.^14^ The real‐world environments and especially hospital environments, usually face a complexity of interactions between several environmental and behavioral factors,^19^ and the air and surface concentrations. A significant positive correlation was previously found between the number of particles with a diameter of >10 μm and the bioaerosol concentration, which leads to an effect on the rate microorganisms that are deposited on open Petri dishes.^20^ Although the diameter of *S. aureus* is known to be about 1 μm, this does not mean that the aerosol in the chamber that carries the *S. aureus* has the same size; the distribution of the nebulizer particle size range is 0.3–5 micron. In a hospital environment, microorganisms, including *Staphylococcus* spp., may be carried on larger particles, such as skin squamae,^21^ which will deposit much more quickly.

Further studies in a hospital environment to quantify the relationship between microorganisms in the air and on surfaces are required with considering the impact of the hospital environment on this relationship. The knowledge that environmental factors have a significant impact on the concentration of microorganisms in the air and on surfaces, and the fact that these factors can be controlled to mitigate the infection risk means that measures can be taken to prevent the level of microorganisms from breaching the accepted threshold by these factors, and by employing efficient cleaning and ventilation systems. A well‐designed ventilation system must also be installed to maintain a healthy environment, and to enable quick recovery in case of a breach of the accepted level of microorganisms.

## 5 CONCLUSIONS

Automated multiplate passive air sampling has been developed as a new sampling device which can enable a sequence of passive deposition samples to be taken through an automated process. The device is a valuable tool for use in controlled environments to provide consistent passive sampling results. It enables the sampling of airborne microorganisms over time, using pre‐configured settings without the need for human intervention. It has particular advantages when used to collect samples in settings where human intervention would either impact the experimental results or is hazardous. AMPAS can provide automated time‐series surface samples and makes it possible to investigate the influence of parameters such as ventilation rate on spatiotemporal bioaerosols. The study has demonstrated the application under steady‐state conditions, but the device can also be used to collect a time‐series under transient conditions due to changing bioaerosol emission rates or ventilation conditions.

Automated multiplate passive air sampling has been used to measure the relationship between microorganisms in the air and on surfaces, which can be represented by the loss rate due to deposition onto surfaces. Increasing the ventilation rate from 3ACH to 6ACH in the chamber reduces the concentration of microorganisms in the air and on surfaces by more than 40%. Since the decrease in concentration occurs in both airborne and deposited microorganisms, the loss rate due to deposition was shown to remain constant as the ventilation rate increases. This means that controlling the ventilation rate can mitigate the infection risk through both air and surface contamination in the indoor environment. Defining a single value for deposition rate is not feasible, but this study, together with previous data, provides a realistic range of values for models and deeper insight into the factors that affect this rate.

## AUTHOR CONTRIBUTIONS

**Waseem Hiwar** contributed to Conception and design of study, acquisition of data, analysis and/or interpretation of data, Drafting the manuscript, Approval of the version of the manuscript to be published. **Marco‐Felipe King** contributed to Conception and design of study, analysis and/or interpretation of data, Drafting the manuscript, Approval of the version of the manuscript to be published. **Harith Kharrufa** contributed to Conception and design of study, Drafting the manuscript, Approval of the version of the manuscript to be published. **Emma Tidswell** contributed to Drafting the manuscript, Approval of the version of the manuscript to be published. **Louise A. Fletcher** contributed to Conception and design of study, Drafting the manuscript, Approval of the version of the manuscript to be published. **CatherineJ. Noakes** contributed to Conception and design of study, analysis and/or interpretation of data, Drafting the manuscript, Approval of the version of the manuscript to be published.

## CONFLICT OF INTEREST

None to declare.

## Data Availability

The data that support the findings of this study are openly available in github at https://github.com/Waseemhiwar/AMPAS‐and‐Ventilation‐paper.git.
